# Impact of oral health problems on daily activities and quality of life of adolescents in Brazil

**DOI:** 10.1590/1980-549720260009.supl.1

**Published:** 2026-07-10

**Authors:** Waneska Ferreira de Melo, Helene Soares Moura, Sandra Aparecida Marinho, Lívia Natália Sales Brito, Gustavo Gomes Agripino, Pierre Andrade Pereira de Oliveira

**Affiliations:** 1 Universidade Estadual da Paraíba – Araruna (PB), Brazil.

**Keywords:** Oral health, Dental caries, Adolescent, Quality of life

## Abstract

**Objective:**

To analyze the impact of oral health conditions on quality of life and daily activities among adolescents according to the Oral Impacts on Daily Performances (OIDP) and its domains.

**Methods:**

This cross-sectional study was based on data from the Brazilian National Oral Health Survey (SB Brasil 2023) and included 8,054 adolescents aged 15 to 19 years from all regions of the country. The outcome was oral health-related quality of life assessed using the OIDP instrument. Associations between OIDP and its domains with the presence of untreated caries, tooth loss, odontalgia, need for prostheses, gingival bleeding, and dental calculus were analyzed. Statistical analyses were performed considering the complex sample design, using the Rao-Scott adjusted F-test and multiple logistic regression considering a 95% confidence interval (CI).

**Results:**

A negative impact of oral health on quality of life was observed in 37.2% of adolescents, with a higher frequency among those with lower family income and education level. All oral health problems analyzed showed a significant association with an OIDP≥1. After adjusting for sociodemographic factors, dental pain in the last 6 months was the condition most strongly associated with a negative impact on quality of life (OR=9.06; 95%CI 6.32–13.0), followed by the need for a prosthesis (OR=3.86; 95%CI 2.31–6.43), untreated dental caries (OR=2.39; 95%CI 1.87–3.05), tooth loss (OR=1.56; 95%CI 1.07–2.26), gum bleeding (OR=1.62; 95%CI 1.14–2.29), and dental calculus (OR=1.33; 95%CI 1.03–1.71).

**Conclusion:**

Oral health problems represent a limiting factor in the quality of life and daily activities of Brazilian adolescents.

## INTRODUCTION

Adolescence is a transitional period between childhood and adulthood, marked by intense biological, psychological, and social transformations, during which individuals become particularly susceptible to risk factors that can generate cumulative impacts^1^. Experiences related to identity formation during adolescence significantly influence the development of behavioral and emotional patterns, with potential repercussions for well-being and life trajectory^2^.

It is common to observe, during adolescence, greater neglect regarding health care^3,4^ and a higher prevalence of behaviors that pose a risk for both oral and systemic diseases^5,6^. In addition to aspects inherent to this developmental stage itself, individual biological factors as well as the socioeconomic and cultural context can influence oral health behaviors^7,8^. Consequently, the occurrence of oral diseases during adolescence is also shaped by the specific context in which the adolescent is embedded^9^.

Although an improvement in the epidemiological landscape of oral health among Brazilian adolescents has been observed over the years^10^, it is important to highlight the heterogeneous distribution of oral health conditions within the population over time — a reflection of socioeconomic and racial inequities across Brazil's various regions^10–13^. This scenario underscores the importance of continuous monitoring of the oral health of Brazilian adolescents, as well as the strengthening of health policies and strategies designed to ensure comprehensiveness and equity in oral health care.

Oral health conditions, such as the presence of untreated dental caries and dental pain, demonstrate a strong correlation with negative impacts on individuals' quality of life; these impacts can encompass physical, psychological, and social dimensions, thereby highlighting the significance of assessing oral health-related quality of life (OHRQoL) within the field of public health^14–16^. This concept, which encompasses the perception of oral health status, functional and emotional well-being, and satisfaction with dental care^16,17^, also bears a direct relationship to socioeconomic characteristics across all age groups^18^.

To assess OHRQoL, the use of specific instruments is essential, such as oral impacts on daily performances (OIDP), a sociodental indicator that has been widely used to evaluate the frequency and severity of oral impacts affecting individuals' daily performance^16,17,19^.

Although studies have evaluated OHRQoL and the impacts of oral health conditions on various aspects of the lives of Brazilian adolescents^20–24^, such studies remain scarce and limited in terms of population and territorial scope, particularly when considering the diversity and vastness of Brazil. This gap hinders a comprehensive understanding of the actual effects of these issues on the quality of life of young Brazilians, as well as the formulation of more effective care strategies for this demographic.

Given that the National Oral Health Survey (SB Brasil) collects demographic and socioeconomic data, along with data on various oral health outcomes and OHRQoL, at both national and specific age-group levels, it has emerged as a key oral health surveillance tool. Furthermore, it serves as a vital data source for assessing the oral health status of Brazilian adolescents, identifying related contextual factors, and examining potential negative consequences associated with oral diseases^10^.

Against this backdrop, the present study aimed to analyze the impact of oral health conditions on the quality of life and daily activities of adolescents aged 15 to 19, utilizing the OIDP index and its specific domains, on the basis of data derived from the SB Brasil 2023 survey.

## METHODS

The present study was a cross-sectional study utilizing secondary data derived from the National Oral Health Survey, namely SB Brasil 2023^10^. As these are secondary data in the public domain, submission to a Research Ethics Committee (CEP) was not required, in accordance with Resolution No. 510/2016 of the National Health Council (CNS).

The survey employed a probabilistic, stratified, and multi-stage cluster sampling design. Sample selection involved households randomly drawn from previously defined census tracts, ensuring proportionality based on population size (capital cities and interior regions). The survey covered 422 municipalities, including the 27 capital cities and various interior cities. Data collection was designed to ensure statistical representativeness at the national, regional, and state levels, encompassing both capital cities and regional interior areas. Additional details regarding the methodology are available in the technical publications of SB Brasil 2023^10,25^.

The sample consisted of 8,054 adolescents aged 15 to 19, who were interviewed and examined in their homes. The dependent variable was constructed based on the impact of oral health on quality of life, assessed using the OIDP index — previously validated for the Brazilian population — which encompasses nine domains investigating difficulties attributed to oral problems in daily activities: eating, speaking, cleaning teeth, sleeping, smiling, studying or working, engaging in leisure activities, maintaining social relationships, and feeling emotionally affected. Possible responses were: "yes," "no," and "don't know."

A global dichotomous variable was created to measure the impact of oral health on quality of life; the presence of at least one affected domain (OIDP≥1) was classified as "with impact," while the absence of any impact across the nine domains (OIDP=0) was classified as "without impact." The association of each domain was also evaluated individually, on the basis of the adolescents' self-reported responses ("yes" = with impact; "no" = without impact).

The independent variables included sociodemographic characteristics (sex, age group, skin color/race, education level, and family income) and oral health conditions (presence of untreated dental caries, tooth loss, toothache, need for prosthetics, gingival bleeding, and presence of dental calculus). Chart 1 details the variables used and the categorization process. The categories "don't know," "didn't answer," and "no information" were treated as missing values and were not included in the statistical analyses.

**Chart 1:** Description of the variables used in the study.

Variable	Original presentation in the SB Brasil 2023 database	Adaptation
OIDP Domains	There are 9 questions regarding dental problems. 0. No; 1. Yes; 9. Don't know*	Unchanged
Global OIDP	Sum of the number of times "Yes" is selected across the 9 questions within the OIDP domains. The numerical value ranges from 0 to 9.	Dichotomization: 0. OIDP=0 1. OIDP≥1
Sex	Participant's sex 1. Male; 2. Female	Unchanged
Skin color/Race	Participant's skin color or race 1. White; 2. Black; 3. Asian; 4. Brown; 5. Indigenous; 9. Don't know/Didn't answer*	Unchanged
Age	Age in full years	Categorized under: 1. 15 to 16 years 2. 17 to 18 years 3. 19 anos
Income	Last month, how much did all the people living in your household receive collectively, in reais, including salaries, Bolsa Família benefits, pensions, rental income, military pay, retirement benefits, or other sources of income? Numeric value in reais.	Categorized according to minimum wage (MW) of 2023 (R$ 1,320.00). 1. Up to 1 MW 2. >1 to 3 MW 3. >3 MW
Education level	Highest level of education/grade/school year completed: 0. No schooling; 1. Literacy training; 2. Incomplete elementary; 3. Completed elementary; 4. Incomplete high school; 5. Completed high school; 6. Incomplete higher education; 7. Completed higher education; 9. Unknown/didn't answer*	Grouped under: 1. Up to elementary 2. High school 3. Higher education
Untreated caries ≥1	Presence of one or more teeth with untreated decay (total decayed ≥1) 0. No decayed teeth; 1. ≥1 decayed tooth	Unchanged
Tooth loss ≥1	Count of the total number of permanent teeth lost in the DMFT index	Dichotomization: 0. No (0) 1. Yes (≥1)
Toothache	In the last 6 months, did you have toothache? 0. No; 1. Yes; 9. Don't know/Didn't answer*	Unchanged
Need for prosthesis	Need for upper and lower dental prostheses. 0. No need for dental prosthesis; 1 to 5: types of prostheses needed. 9. No information*	Dichotomization: 0. Not needed 1. Needed
Gum bleeding/ Presence of calculus	Bleeding/calculus 0. Remaining sextants healthy or showing another periodontal condition 1. 1 to 6 sextants with bleeding on probing/with dental calculus	Category name simplification 0. No 1. Yes

* Data corresponding to the responses "I don't know," "didn't answer," and "no information" were treated as missing data in the database;

DMFT: decayed, missing, filled – permanent teeth.; MW: minimum wage.

Statistical analyses were performed using the Statistical Package for the Social Sciences (SPSS®) software, version 20, using the complex samples module and accounting for the sample weights present in the dataset. Initially, a descriptive analysis of the variables was conducted, involving the calculation of weighted proportions. Subsequently, bivariate analyses were performed to investigate associations between OIDP (both globally and by domain) and the independent variables, using the Rao-Scott independence test — an adjusted version of Pearson's χ^2^ test designed for complex samples.

**Figure 1:**
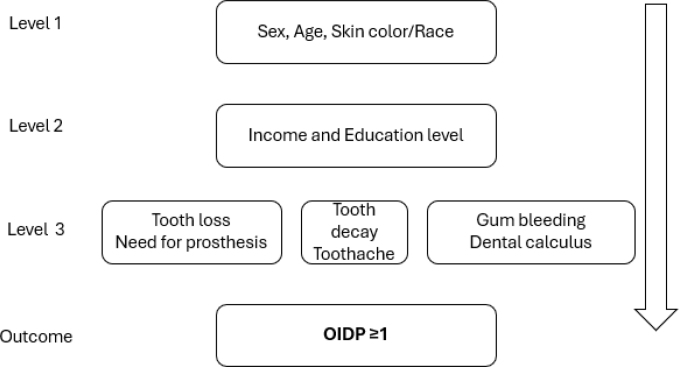
Theoretical model of the association between independent variables and quality of life.

Multiple logistic regression was based on a theoretical model using a hierarchical approach^26^, wherein the first level included demographic variables (sex, age, and skin color/race), the second level consisted of socioeconomic variables (income and education level), and the third level encompassed oral health conditions (Figure 1). Initially, unadjusted odds ratios (ORs) were calculated for all independent variables. Only those variables that yielded p<0.20 in the bivariate analysis were included in the adjusted models. Following adjustment, only independent variables with a p<0.20 remained in the final model. The final models estimated adjusted ORs, along with their respective 95% confidence interval (CI), adopting a significance level of 5% (p<0.05).

To minimize the influence of variables exhibiting conceptual or statistical interrelationships, the adjusted models were structured into three groups of oral health conditions; this approach served to reduce redundancy among predictors and enhance model stability.

Multicollinearity among the independent variables included in the final models was assessed using tolerance values, the variance inflation factor (VIF), and condition indices. No values indicative of collinearity were observed, with tolerance values ranging from 0.64 to 0.99, VIF values between 1.01 and 1.57, and a maximum condition index of 16.33. These results indicate an absence of multicollinearity, allowing the variables to remain in the models.

### Data Availability Statement:

The entire dataset supporting the results of this study is made available within the article itself.

## RESULTS

The sample consisted of adolescents of both sexes, with a slight male predominance (50.2%), a majority self-identifying as brown (47.9%), and who reported a family income of one to three minimum wages (49.0%). It was found that 43.7% had one or more decayed teeth, 13.5% had lost at least one tooth due to caries, toothache within the previous six months was reported by 21.2% of the adolescents, 9.3% required a prosthesis, 35.3% showed dental calculus, and 34.4% presented with gum bleeding (Table 1).

**Table 1:** Association between socioeconomic variables, oral health conditions, and Oral Impacts on Daily Performance (OIDP)≥1 among Brazilian adolescents aged 15 to 19.

Variable	Category	Sample		OIDP≥1	p-value
		n	%	% (95%CI)	
General		8,054	100	37.2	-
Sex	Male	3,875	50.2	34.4 (30.3–38.7)	0.055
	Female	4,178	49.8	40.0 (34.7–45.5)	
Skin color/Race	White	2,604	38.0	34.8 (27.5–42.8)	0.390
	Black	1,028	12.5	40.8 (32.9–49.3)	
	Asian	107	1.3	35.9 (21.5–53.4)	
	Brown	4,205	47.9	38.2 (33.9–42.6)	
	Indigenous	19	0.4	7.8 (1.8–28.5)	
Age (years)	15–16	3,874	43.2	36.2 (31.9–40.8)	0.594
	17–18	2,751	39.6	36.6 (30.2–43.6)	
	19	1,428	17.2	40.8 (33.3–48.8)	
Income (MW)	Up to 1	1,474	30.7	46.4 (39.0–53.9)	0.004*
	>1 to 3	2,517	49.0	38.1 (30.8–45.8)	
	>3	1,027	20.3	27.2 (21.3–34.1)	
Education level	Up to elem.	2,053	23.1	43.9 (38.1–49.9)	0.010*
	High school	5,434	71.5	35.9 (31.2–40.8)	
	Higher ed.	479	5.4	26.3 (18.9–35.4)	
Untreated caries	≥1 tooth	3,410	43.7	49.6 (43.9–55.3)	<0.001*
	No	4,631	56.3	27.6 (24.4–31.2)	
Tooth loss	≥1 tooth	1,146	13.5	53.7 (45.6–61.7)	<0.001*
	No	6,895	86.5	34.6 (30.9–38.5)	
Toothache	Yes	1,708	21.2	76.1 (69.0–82.0)	<0.001*
	No	6,289	78.8	26.5 (23.3–29.9)	
Need for prosthesis	Yes	836	9.3	67.2 (57.8–74.4)	<0.001*
	No	7,205	90.7	34.1 (30.5–38.0)	
Gum bleeding	Yes	2,707	34.4	46.3 (40.9–51.8)	<0.001*
	No	5,334	65.6	32.4 (27.9–37.3)	
Presence of calculus	Yes	2,940	35.3	42.8 (37.4–48.4)	0.003*
	No	5,101	64.7	34.2 (30.0–38.6)	

Test of independence with Rao-Scott correction for complex samples; Sample: absolute number (unweighted) of individuals per category;

* p<0.05: considered significant; 95%CI: 95% confidence interval; OIDP: Oral Impacts on Daily Performances; MW: minimum wage.

Table 1 reveals an impairment in quality of life among 76.1% of those reporting toothache within the last six months; 67.2% of those requiring dental prostheses; 53.7% of those presenting with at least one missing tooth; 49.6% of those with one or more decayed teeth; 46.3% of those presenting with gingival bleeding; and 42.8% of those with the presence of dental calculus. A greater impairment of quality of life was also observed among individuals with a family income of up to one minimum wage (46.4%).

Table 2 presents the association between oral health conditions and the daily activities of adolescents. It is observed that having one or more decayed teeth, one or more missing teeth, a history of toothache, and the need for a prosthesis were associated with all three dimensions evaluated — physical/functional, psychological/emotional, and social — whereas the presence of dental calculus showed no association with any of these dimensions.

**Table 2:** Association between dental caries, tooth loss, toothache, need for prosthesis, gum bleeding, and presence of calculus in daily activities among Brazilian adolescents aged 15 to 19.

OIDP Domain		≥1 decayed tooth	≥1 lost tooth	Toothache	Need for prosthesis	Gum bleeding	Presence of calculus
		% (95%CI)	% (95%CI)	% (95%CI)	% (95%CI)	% (95%CI)	% (95%CI)
Physical/functional							
	Difficulty eating	30.0 (25.2–35.2)*	32.4 (24.6–41.3)*	52.0 (44.1–59.9)*	46.1 (36.9–55.6)*	24.6 (20.4–29.5)**	21.3 (17.4–25.9)
	Difficulty speaking	9.0 (6.3–12.6)*	13.6 (8.2–21.5)*	18.3 (12.7–25.7)*	18.8 (11.3–29.7)*	6.3 (4.1–9.6)	4.7 (3.3–6.7)
	Discomfort when brushing	16.3 (12.4–21.2)	20.7 (14.5–28.9)**	16.6 (11.6–23.2)	18.7 (11.6–28.8)	14.8 (11.7–18.6)	16.2 (12.2–21.0)
	Stopped playing sports	5.9 (4.4–7.9)*	9.2 (6.1–13.6)*	10.9 (7.7–15.2)*	11.3 (7.2–17.1)*	4.0 (2.8–5.5)	3.7 (2.7–5.0)
Psychological /emotional							
	Felt nervous or irritable	25.7 (21.3–30.7)*	31.7 (24.1–40.5)*	46.6 (40.0–53.5)*	43.3 (32.5–54.9)*	19.5 (15.5–24.2)	17.6 (14.0–21.9)
	Slept poorly/stopped sleeping	30.0 (25.7–34.6)*	37.6 (29.5–46.4)*	52.3 (43.7–60.8)*	45.3 (35.9–55.1)*	21.9 (18.1–26.1)**	19.4 (15.9–23.6)
	Felt embarrassed to smile or speak	21.0 (16.8–25.8)*	18.7 (12.9–26.2)	33.1 (27.0–39.9)*	25.5 (19.2–33.1)**	20.5 (16.7–24.9)**	19.4 (15.0–24.8)
Social							
	Stopped going out/having fun/going to parties	11.1 (8.6–14.3)*	16.9 (11.4–24.4)*	21.8 (17.0–27.5)*	24.0 (16.2–34.1)*	7.8 (5.9–10.2)	7.0 (5.3–9.2)
	It got in the way of studying/working/chores	10.6 (7.8–14.3)*	16.2 (10.5–24.1)*	20.9 (15.4–27.9)*	16.6 (10.3–25.6)*	6.1 (4.7–8.0)	6.6 (4.5–9.8)

Test of independence with Rao-Scott correction for complex samples;

* p≤0.001;

** p<0.05: considered significant. Comparison between the groups with and without the adverse event; 95%CI: 95% confidence interval; OIDP: Oral Impacts on Daily Performances.

Having one or more decayed teeth was associated (p<0.001) with difficulty eating (30.0%), sleep disturbances (30.0%), and nervousness/irritability (25.7%). Tooth loss was associated (p<0.001) with sleep disturbances (37.6%), difficulty eating (32.4%), and nervousness/irritability (31.7%). Regarding toothache, more than half (52.0%) of the affected adolescents reported difficulty eating and experienced sleep disturbances (52.3%), while 46.6% felt nervous or irritable, with a statistically significant difference (p<0.001). The need for a dental prosthesis was associated (p<0.001) with difficulty eating (46.1%), sleep disturbances (45.3%), and nervousness/irritability (43.3%). Among adolescents with gingival bleeding, 24.6% reported difficulty eating (p=0.005); 21.9% experienced sleep disturbances (p=0.029); and 20.5% felt embarrassed to smile or speak (p=0.017) (Table 2).

In unadjusted logistic regression analysis, associations were observed between OIDP≥1 and income, education level, and oral health conditions. Adolescents with lower income were 2.31 times more likely to report an impact on quality of life, while those with lower education level were 2.19 times more likely. Among the oral health conditions, all were associated with the outcome, with dental pain being the most significant factor, increasing the odds of OIDP≥1 by nearly nine times. The need for prostheses also demonstrated a strong association, increasing the odds of impact by nearly four times. Furthermore, the presence of untreated caries raised the odds by 2.57 times, tooth loss by 2.20 times, gingival bleeding by 1.80 times, and dental calculus by 1.44 times, as shown in Table 3.

**Table 3:** Logistic regression model (unadjusted) for the presence of Oral Impacts on Daily Performances (OIDP)≥1, associated with sociodemographic variables and oral health conditions, among Brazilian adolescents aged 15 to 19.

Variable	Category compared	OR	95%CI	p-value
Sex	Female *vs*. Male	1.27	0.99–1.63	0.055
Skin color/Race	Black vs. White	1.29	0.84–1.99	0.436
	Asian *vs*. White	1.05	0.51–2.18	
	Brown *vs*. White	1.16	0.77–1.73	
	Indigenous *vs*. White	0.16	0.03–0.78	
Age (years)	17–18 *vs*. 15–16	1.02	0.74–1.39	0.595
	19 *vs*. 15–16	1.21	0.85–1.74	
Family income (MW)	≤1 *vs*. >3	2.31	1.41–3.79	0.005*
	1–3 *vs*. >3	1.64	1.04–2.60	
Education level	Up to primary *vs*. Higher ed.	2.19	1.34–3.58	0.010*
	High school *vs*. Higher ed.	1.56	1.01–2.43	
Untreated caries ≥1	Yes *vs*. No	2.57	2.05–3.23	<0.001*
Tooth loss ≥1	Yes *vs*. No	2.20	1.62–2.97	<0.001*
Pain in last 6 months	Yes *vs*. No	8.85	6.48–12.10	<0.001*
Need for prosthesis	Yes *vs*. No	3.96	2.65–5.91	<0.001*
Gum bleeding	Yes *vs*. No	1.80	1.36–2.38	<0.001*
Presence of calculus	Yes *vs*. No	1.44	1.13–1.84	0.003*

* p<0.05 considered significant; OR: odds ratio; 95%CI: 95% confidence interval; OIDP: Oral Impacts on Daily Performances.

In the adjusted models, the variables of income and education lost significance; however, oral health conditions maintained a strong association with OIDP≥1. In Model 1, adolescents with untreated caries had 2.39 times higher odds of experiencing an impact, while those with dental calculus showed a 1.33-fold increase in odds. In Model 2, dental pain remained the condition most strongly associated with the outcome, increasing the odds by more than nine times, whereas tooth loss raised the odds by 1.56. In Model 3, the need for prostheses stood out, increasing the odds of OIDP≥1 by nearly four times, and gum bleeding increased the odds by 1.62. The various oral health conditions maintained a significant association with the adolescents' quality of life, even after adjusting for sociodemographic factors (Table 4).

**Table 4:** Logistic regression model for Oral Impacts on Daily Performances (OIDP) ≥1, adjusted for sex, income, education level, and oral health conditions, in Brazilian adolescents aged 15 to 19.

Variables		Model 1		Model 2		Model 3	
		OIDP≥1 adjusted for sex, income, education level, untreated caries, and presence of calculus		OIDP≥1 adjusted for sex, income, education level, and tooth pain and loss		OIDP≥1 adjusted for sex, income, education level, need for prosthesis, and gum bleeding	
		OR (95%CI)	p-value	OR (95%CI)	p-value	OR (95%CI)	p-value
Sex			0.064		0.093		0.014*
	Female *vs*. Male	1.28 (0.98–1.67)		1.38 (0.94–2.03)		1.55 (1.09–2.20)	
Income (MW)			a		0.184		0.083
	≤1 *vs*. >3	a		1.63 (0.96–2.78)		1.79 (1.10–2.92)	
	1–3 *vs*. >3	a		1.29 (0.78–2.11)		1.40 (0.86–2.29)	
Education level			0.055		0.190		0.081
	Up to elementary *vs*. Higher education	1.76 (1.08–2.86)		1.97 (1.01–3.84)		2.30 (1.11–4.77)	
	High school *vs*. Higher ed.	1.33 (0.86–2.06)		1.70 (0.95–3.07)		1.84 (0.96–3.52)	
Untreated caries (≥1)		2.39 (1.87–3.05)	<0.001*	–		–	
Pain in last 6 months		–		9.06 (6.32–13.0)	<0.001*	–	
Tooth loss ≥1		–		1.56 (1.07–2.26)	0.019*	–	
Need for prosthesis		–		–		3.86 (2.31–6.43)	<0.001*
Gum bleeding		–		–		1.62 (1.14–2.29)	0.006*
Presence of calculus		1.33 (1.03–1.71)	0.027*	–		–	

* p<0.05: statistically significant;

a Excluded after entry into model, p>0.20 in adjusted analysis; OR: odds ratio; 95%CI: 95% confidence interval; MW: minimum wage; OIDP: Oral Impacts on Daily Performances.

## DISCUSSION

The results of this study revealed a significant association between nearly all the analyzed variables regarding oral health conditions and the quality of life of Brazilian adolescents. These findings align with the study by Peres et al.^27^ who, upon analyzing adolescents aged 15 to 19 participating in the SB Brasil 2010 survey, identified that the presence of toothache, tooth loss, and untreated carious lesions was associated with significant impacts on quality of life.

Toothache was the condition that most impacted the adolescents' quality of life, being primarily associated with eating, sleep, and irritability. It serves as a significant public health marker, frequently used to assess the consequences and particularly of dental caries, and it is also considered one of the primary motivations for seeking care, as well as a reflection of the conditions regarding access to oral health services^28,29^.

Given that it affects various aspects related to the quality of life of children and adolescents^14^, toothache is of particular concern during the school years, as it is associated with impaired performance and school absenteeism and is considered an indicator of distress and social vulnerability^30^.

Untreated dental caries, commonly the cause of toothache, showed a high prevalence among adolescents, impacting all dimensions assessed by OIDP; this finding corroborates a meta-analysis that highlighted the negative impact of carious lesions on the daily activities of adolescents^31^. Reinforcing this relationship, a recent literature review demonstrated that the greater the severity of carious lesions, the greater the impairment of oral health-related quality of life in adolescents^15^.

As noted by Gerritsen et al.^32^ in a meta-analysis, tooth loss is also associated with unfavorable scores regarding oral health-related quality of life (OHRQoL)^32^. Furthermore, among the adolescents surveyed in the SB Brasil 2023 study, it was observed that the loss of at least one tooth impacted various domains related to the evaluated outcome, specifically sleep disturbances, difficulties with eating, and emotional changes such as nervousness or irritability.

As a consequence of tooth loss, it was observed that 9.3% of adolescents required dental prostheses; although this represents a low prevalence, it warrants greater attention given that it concerns such a young population. It is crucial to recognize that tooth loss occurring during adolescence constitutes a serious issue, as there is a tendency for it to progressively worsen throughout an individual's lifetime^33^, particularly when considering the chronic nature of the primary oral diseases that lead to tooth loss. It is important to emphasize that uncompensated tooth loss can substantially compromise the physical and psychological well-being of young people, as identified by the analyses in the present study, thereby underscoring the critical importance of rehabilitative interventions for this age group.

Of the periodontal changes investigated, only gingival bleeding affected the adolescents' daily activities, albeit to a lesser degree compared to other health issues. Some studies have identified an association between the presence of gingivitis and dental calculus and impacts on smiling, school performance, and social interactions at age 12^34^, which were also linked to impairments in smile aesthetics at age 15^25^. However, Ripardo et al.^35^ highlight that, although periodontal diseases are common during adolescence, there is a low perception of their severity, making it difficult to establish a direct link between these conditions and impairments in daily life.

The observed profile — characterized by a high prevalence of untreated caries among adolescents whose OHRQoL is primarily impacted by toothache and the need for prostheses — reveals potential weaknesses in preventive measures targeting this demographic, as well as in early diagnosis and continuity of care. A lack of treatment during the early stages can trigger a cumulative effect, leading to disease progression, increased pain, and in many cases tooth loss, thereby progressively compromising the adolescents' well-being.

Notwithstanding the attention currently directed toward adolescent health care, a revision of care strategies; including more effective intersectoral initiatives, is deemed necessary. Strengthening the role of oral health teams within the scope of the Health in School Program (Programa Saúde na Escola), for instance, presents a strategic opportunity for promoting and protecting oral health, given that a significant portion of this age group remains within the school environment. The implementation of educational and preventive measures, along with initiatives for the early detection of health issues specifically tailored to the unique needs of adolescents, could contribute significantly to improving the observed oral health landscape, thereby yielding positive repercussions on the quality of life of this population.

Regarding the sociodemographic variables assessed, it was observed that girls were more likely to report a negative impact of oral health on their quality of life — particularly within the statistical model adjusted for the need for prostheses and gingival bleeding. Similar results were identified by Santos et al.^28^, in a systematic review with meta-analysis, in which girls showed higher odds of reporting toothache, and by Militi et al.^36^, who identified a greater impact of dental appearance on girls' psychological well-being and self-confidence. This may indicate heightened self-perception and, consequently, a greater likelihood of self-reporting the impact of oral health on quality of life, particularly during adolescence.

Although the variables of income and education did not show an association with OIDP in the adjusted models, this result does not invalidate their social relevance. The literature consistently demonstrates a relationship between poorer levels of these variables and lower levels of oral health literacy, poorer self-care habits, reduced access to dental services, and a higher incidence of oral diseases^7–9,37^, suggesting that income and education may influence OHRQoL more indirectly, mediated by the presence of clinical conditions and the utilization of health services. Furthermore, in the Brazilian context, the provision of public oral health services may partially attenuate the direct effect of income on self-reported impact, without however eliminating the underlying social inequalities.

This study highlighted the impacts of oral health conditions on the quality of life of adolescents at a national level; however, certain limitations must be considered. The cross-sectional design precluded the establishment of causal relationships between the analyzed variables, thereby restricting inferences to statistical associations. Furthermore, data regarding quality of life were obtained via self-report, which may be subject to recall bias, as well as the underestimation or overestimation of symptoms.

Consequently, it is recommended that future studies adopt longitudinal designs capable of tracking the progression of oral health conditions over time and their cumulative repercussions on quality of life. Qualitative approaches could deepen the understanding of adolescents' subjective experiences regarding the limitations imposed by oral health problems, thereby contributing to the development of more sensitive and integrated care strategies. Investigations that incorporate social determinants of health, such as education level, geographic location, race/skin color, and access to public policies, are equally relevant for enhancing intersectoral initiatives aimed at promoting adolescent oral health across diverse sociocultural contexts.

In summary, the findings of this study broaden our understanding of the primary oral health conditions affecting Brazilian adolescents and their implications for quality of life. These findings provide health managers with epidemiological evidence that can inform the planning, monitoring, and rational allocation of resources, thereby strengthening oral health surveillance within the framework of the National Oral Health Policy. For oral health teams, these findings help in identifying priority health conditions, enhancing risk stratification, scheduling organization, and the targeting of preventive, diagnostic, and resolution-oriented measures in the country.
