# Supplementary material for: Impact of oral health problems on daily activities and quality of life of adolescents in Brazil
Source: Rev Bras Epidemiol. 2026 Jul 10;29(Suppl 1):e260009supl1. doi: 10.1590/1980-549720260009.supl.1 (PMC13360982; doi:10.1590/1980-549720260009.supl.1)
Supplement: Translation

# Translation: Impacto dos agravos em saúde bucal nas atividades diárias e qualidade de vida de adolescentes do Brasil

**DOI:** 10.1590/1980-549720260009.supl.1.2

**Authors:** Waneska Ferreira de Melo, Helene Soares Moura, Sandra Aparecida Marinho, Lívia Natália Sales Brito, Gustavo Gomes Agripino, Pierre Andrade Pereira de Oliveira

**Affiliations:** I Universidade Estadual da Paraíba – Araruna (PB), Brasil.

**Keywords:** Saúde bucal, Cárie dentária, Adolescente, Qualidade de vida

## Abstract

**Objetivo:**

Analisar o impacto dos agravos em saúde bucal na qualidade de vida e nas atividades diárias de adolescentes conforme o *Oral Impacts on Daily Performances* (OIDP) e seus domínios.

**Métodos:**

Trata-se de um estudo transversal baseado em dados da Pesquisa Nacional de Saúde Bucal (SB Brasil 2023) envolvendo 8.054 adolescentes de 15 a 19 anos de todas as regiões do país. O desfecho foi a qualidade de vida relacionada à saúde bucal avaliada pelo instrumento OIDP. Foram analisadas as associações entre o OIDP e seus domínios com a presença de cárie não tratada, perda dentária, odontalgia, necessidade de prótese, sangramento gengival e cálculo dental. As análises estatísticas foram realizadas considerando-se o desenho amostral complexo, utilizando-se o teste F ajustado de Rao-Scott e de regressão logística múltipla com intervalo de confiança (IC) de 95%.

**Resultados:**

Observou-se impacto negativo da saúde bucal na qualidade de vida em 37,2% dos adolescentes, com maior frequência entre aqueles de menor renda familiar e escolaridade. Todos os agravos bucais analisados apresentaram associação significativa com o OIDP≥1. Após ajuste por fatores sociodemográficos, a dor dentária nos últimos seis meses foi o agravo mais fortemente associado ao impacto negativo na qualidade de vida (*odds ratio* — OR=9,06; IC95% 6,32–13,0), seguida da necessidade de prótese (OR=3,86; IC95% 2,31–6,43), cárie dentária não tratada (OR=2,39; IC95% 1,87–3,05), perda dentária (OR=1,56; IC95% 1,07–2,26), sangramento gengival (OR=1,62; IC95% 1,14–2,29) e cálculo dentário (OR=1,33; IC95% 1,03–1,71).

**Conclusão:**

Os agravos bucais representam um fator limitante na qualidade de vida e nas atividades diárias dos adolescentes brasileiros.

## INTRODUÇÃO

A adolescência é um período de transição entre a infância e a vida adulta marcado por intensas transformações biológicas, psicológicas e sociais, no qual os indivíduos se tornam especialmente suscetíveis a fatores de risco que podem gerar impactos cumulativos^1^. As experiências relacionadas à formação da identidade na adolescência influenciam significativamente o desenvolvimento de padrões comportamentais e emocionais, com possíveis repercussões no bem-estar e na trajetória de vida^2^.

É comum ser observada na adolescência maior negligência nos cuidados com a saúde^3,4^ e maior prevalência de comportamentos de risco para doenças bucais e sistêmicas^5,6^. Além de aspectos inerentes à própria fase, fatores biológicos individuais e o contexto socioeconômico e cultural podem influenciar os comportamentos de saúde bucal^7,8^. Por sua vez, a ocorrência de doenças bucais na adolescência também será marcada por esse contexto em que o adolescente se encontra inserido^9^.

Embora tenha sido observado ao longo dos anos uma melhoria no panorama epidemiológico da saúde bucal de adolescentes brasileiros^10^, é importante ressaltar a característica de distribuição heterogênea dos agravos à saúde bucal ao longo do tempo na população como reflexo das iniquidades socioeconômicas e raciais nas diferentes regiões brasileiras^10–13^. Este cenário reforça a importância do monitoramento contínuo da saúde bucal dos adolescentes brasileiros, bem como do fortalecimento de políticas de saúde e de estratégias que garantam integralidade e equidade na atenção à saúde bucal.

Agravos bucais, como a presença de cárie não tratada e dor dentária, apresentam forte correlação com impactos negativos na qualidade de vida dos indivíduos, podendo abranger aspectos físicos, psicológicos e sociais, destacando-se assim no campo da saúde pública a avaliação da Qualidade de Vida Relacionada à Saúde Bucal (QVRSB)^14–16^. Esse conceito, que envolve a percepção do estado de saúde bucal, o bem-estar funcional e emocional e a satisfação com a assistência odontológica^16,17^, também tem relação direta com as características socioeconômicas em todas as faixas etárias^18^.

Para a avaliação da QVRSB é fundamental o uso de instrumentos específicos, a exemplo do *Oral Impacts on Daily Performances* (OIDP), considerado um indicador sociodental que tem sido amplamente empregado na avaliação da frequência e da severidade dos impactos bucais que afetam o desempenho diário dos indivíduos^16,17,19^.

Embora existam estudos que avaliaram a QVRSB e os impactos dos agravos bucais nos diferentes aspectos da vida de adolescentes brasileiros^20–24^, eles ainda são escassos e limitados do ponto de vista de amplitude populacional e territorial ao se considerar a diversidade e extensão do território brasileiro. Tal lacuna limita a compreensão dos efeitos reais desses problemas na qualidade de vida dos jovens brasileiros e a formulação de estratégias de cuidado mais eficientes para esse grupo.

Uma vez que a Pesquisa Nacional de Saúde Bucal (SB Brasil) coleta dados demográficos, socioeconômicos de diversos desfechos de saúde bucal e da QVRSB em nível nacional e em diferentes faixas etárias, tornou-se uma ferramenta de vigilância em saúde bucal e é uma importante fonte de dados para avaliar a qualidade de saúde bucal de adolescentes brasileiros, fatores contextuais relacionados, além de possíveis consequências negativas relacionadas às doenças bucais^10^.

Diante desse contexto, o presente estudo teve como objetivo analisar o impacto dos agravos em saúde bucal na qualidade de vida e nas atividades diárias de adolescentes de 15 a 19 anos conforme o OIDP e seus domínios com base nos dados do SB Brasil 2023.

## MéTODOS

O presente estudo é do tipo transversal, com utilização de dados secundários provenientes da Pesquisa Nacional de Saúde Bucal — SB Brasil 2023^10^. Por se tratar de dados secundários de domínio público, não houve necessidade de submissão ao Comitê de Ética em Pesquisa (CEP), conforme a Resolução do Conselho Nacional de Saúde (CNS) n. 510/2016.

A amostragem do inquérito foi do tipo probabilística, estratificada e por conglomerados em múltiplos estágios. A seleção amostral considerou domicílios sorteados em setores censitários previamente definidos, respeitando a proporcionalidade por porte populacional (capitais e interior). A pesquisa abrangeu 422 municípios, incluindo as 27 capitais e cidades do interior. A coleta de dados foi desenhada para garantir representatividade estatística nacional, regional e estadual das capitais e dos interiores regionais. Detalhes adicionais da metodologia estão disponíveis nas publicações técnicas do SB Brasil 2023^10,25^.

A amostra foi composta de 8.054 adolescentes de 15 a 19 anos, entrevistados e examinados em seus domicílios.

A variável dependente foi construída com base no impacto da saúde bucal na qualidade de vida avaliado por meio do índice OIDP, previamente validado para a população brasileira, que contempla nove domínios que investigam dificuldades atribuídas a problemas bucais nas atividades cotidianas: comer, falar, higienizar os dentes, dormir, sorrir, estudar ou trabalhar, praticar atividades de lazer, manter relações sociais e sentir-se emocionalmente afetado. Respostas possíveis: "sim", "não" e "não sabe".

Foi criada uma variável dicotômica global para mensurar o impacto da saúde bucal na qualidade de vida, considerando-se como "com impacto" a presença de pelo menos um domínio afetado (OIDP≥1) e "sem impacto" a ausência de qualquer impacto nos nove domínios (OIDP=0). Também foi avaliada a associação de cada domínio individualmente, com base nas respostas autorreferidas dos adolescentes ("sim"=com impacto; "não"=sem impacto).

As variáveis independentes incluíram características sociodemográficas (sexo, faixa etária, cor/raça, escolaridade e renda familiar) e os agravos bucais (presença de cárie dentária não tratada, perda dentária, dor de dente, necessidade de prótese, sangramento gengival e presença de cálculo dentário). O Quadro 1 detalha as variáveis utilizadas e o processo de categorização. As categorias "não sei", "não respondeu" e "sem informação" foram tratadas como valores perdidos, não sendo incluídas nas análises estatísticas.

**Quadro 1:** Descrição das variáveis utilizadas no estudo.

Variável	Apresentação original no banco do SB Brasil 2023	Adaptação
OIDP Domínios	São 9 questionamentos sobre os problemas com dentes. 0. Não; 1. Sim; 9. Não sabe*	Sem modificação
OIDP Global	Soma de quantas vezes o sim é respondido entre as 9 questões dos domínios do OIDP. Valor numérico varia de 0 a 9.	Dicotomização: 0. OIDP=0 1. OIDP≥1
Sexo	Sexo do participante 1. Masculino; 2. Feminino	Sem modificação
Cor/Raça	Qual a cor ou raça do participante 1. Branca; 2. Preta; 3. Amarela; 4. Parda; 5. Indígena; 9. Não sabe/não respondeu*	Sem modificação
Idade	Idade em anos completos	Categorizado em: 1. 15 a 16 anos 2. 17 a 18 anos 3. 19 anos
Renda	No mês passado, quanto receberam, em reais, juntas, todas as pessoas que moram na sua casa, incluindo salários, bolsa família, pensão, aluguel, soldo, aposentadoria, ou outros rendimentos. Valor numérico em reais.	Categorizado com base no salário mínimo (SM) de 2023 (R$ 1.320,00). 1. Até 1 SM 2.>1 a 3 SM 3.>3 SM
Escolaridade	Qual curso/Série/ano escolar mais elevado: 0. Não estudou; 1. Alfabetização; 2. Fund. incompleto; 3. Fund. completo; 4. Médio inc.; 5. Médio compl.; 6. Superior inc.; 7. Superior compl.; 9. Não sei/não respondeu*	Agrupado em: 1. Até Fundamental 2. Ensino Médio 3. Superior
Cárie não tratada ≥1	Presença de um ou mais dentes com cárie não tratada (cariado total>=1) 0. Sem cariados; 1.>= 1 dente cariado	Sem modificação
Perda dentária ≥1	Contagem do número total de dentes permanentes perdidos no CPO-D	Dicotomização 0. Não (0) 1. Sim (≥1)
Dor de dente	Nos últimos 6 meses, teve dor de dente 0. Não; 1. Sim; 9. Não sabe/não respondeu*	Sem modificação
Necessidade de prótese	Necessidade de prótese dentária sup. e inf. 0. Não necessita prótese dentária; 1 a 5 tipos de próteses que necessita. 9. Sem informação*	Dicotomização: 0. Não necessita 1. Necessita
Sangramento gengival/ Presença de cálculo	Sangramento/ cálculo 0. Demais sextantes hígidos ou com outra condição periodontal 1. 1 a 6 sextantes com sangramento à sondagem/ com cálculo dentário	Simplificação do nome da categoria 0. Não 1. Sim

* Os dados com respostas não sei, não respondeu e sem informação foram tratados como dados perdidos no banco;

CPO-D: Cariados, perdidos, obturados – dente permanente; SM: salário-mínimo.

As análises estatísticas foram realizadas no *software* *Statistical Package for the Social Sciences* (SPSS^®^) versão 20, utilizando o módulo de amostragem complexa, considerando os pesos amostrais presentes no banco. Inicialmente, foi realizada a análise descritiva das variáveis, com cálculo das proporções ponderadas. Em seguida, foram conduzidas análises bivariadas para investigar associações entre o OIDP (global e por domínio) e as variáveis independentes, por meio do teste de independência de Rao-Scott, uma versão ajustada do teste χ^2^ de Pearson para amostras complexas.

A regressão logística múltipla foi baseada em um modelo teórico com abordagem hierarquizada^26^, no qual o primeiro nível incluiu variáveis demográficas (sexo, idade e cor/raça), o segundo foi composto de variáveis socioeconômicas (renda e escolaridade), e o terceiro nível incluiu os agravos bucais (Figura 1). Inicialmente foram calculados os *odds ratios* (OR) de forma não ajustada para todas as variáveis independentes. Só foram incluídas nos modelos ajustados as variáveis que apresentaram p<0,20 na análise bivariada. Após o ajuste, permaneceram no modelo final apenas as variáveis independentes com valor de p<0,20. Os modelos finais estimaram OR ajustados, com respectivos intervalos de confiança (IC) de 95%, adotando-se nível de significância de 5% (p<0,05).

**Figura 1:** Modelo teórico de associação entre variáveis independentes e qualidade de vida.

Para minimizar a influência de variáveis com relação conceitual ou estatística entre si, os modelos ajustados foram estruturados em três grupos de agravos, o que permitiu reduzir a redundância entre preditores e melhorar a estabilidade dos modelos.

A multicolinearidade entre as variáveis independentes incluídas nos modelos finais foi avaliada por meio da tolerância, do fator de inflação da variância (VIF) e dos índices de condição. Não foram observados valores indicativos de colinearidade, com tolerância variando entre 0,64 e 0,99, VIF entre 1,01 e 1,57 e maior índice de condição de 16,33. Esses resultados indicam ausência de multicolinearidade, permitindo a permanência das variáveis nos modelos.

### Declaração de disponibilidade de dados:

Todo o conjunto de dados que sustenta os resultados deste estudo encontra-se disponibilizado no próprio artigo.

## RESULTADOS

A amostra foi composta de adolescentes de ambos os sexos, com ligeira predominância do sexo masculino (50,2%), maioria autodeclarada de cor parda (47,9%) e que afirmou apresentar renda familiar de um a três salários-mínimos (49,0%). Foi identificado que 43,7% apresentaram um ou mais dentes cariados, 13,5% perderam ao menos um dente em decorrência de cárie, a odontalgia nos últimos seis meses foi mencionada por 21,2% dos adolescentes, 9,3% necessitavam de prótese, 35,3% dos adolescentes apresentaram cálculo e 34,4% apresentaram sangramento gengival (Tabela 1).

**Tabela 1:** Associação entre variáveis socioeconômicas, agravos em saúde bucal e *Oral Impacts on Daily Performance* (OIDP)≥1 em adolescentes brasileiros de 15 a 19 anos.

Variável	Categoria	Amostra		OIDP≥1	Valor p
		n	%	% (IC95%)	
Geral		8.054	100	37,2	-
Sexo	Masculino	3.875	50,2	34,4 (30,3–38,7)	0,055
	Feminino	4.178	49,8	40,0 (34,7–45,5)	
Cor/Raça	Branca	2.604	38,0	34,8 (27,5–42,8)	0,390
	Preta	1.028	12,5	40,8 (32,9–49,3)	
	Amarela	107	1,3	35,9 (21,5–53,4)	
	Parda	4.205	47,9	38,2 (33,9–42,6)	
	Indígena	19	0,4	7,8 (1,8–28,5)	
Idade (anos)	15–16	3.874	43,2	36,2 (31,9–40,8)	0,594
	17–18	2.751	39,6	36,6 (30,2–43,6)	
	19	1.428	17,2	40,8 (33,3–48,8)	
Renda (SM)	Até 1	1.474	30,7	46,4 (39,0–53,9)	0,004*
	>1 a 3	2.517	49,0	38,1 (30,8–45,8)	
	>3	1.027	20,3	27,2 (21,3–34,1)	
Escolaridade	Até Fundam.	2053	23,1	43,9 (38,1–49,9)	0,010*
	Médio	5434	71,5	35,9 (31,2–40,8)	
	Superior	479	5,4	26,3 (18,9–35,4)	
Cárie não tratada	≥1 dente	3.410	43,7	49,6 (43,9–55,3)	<0,001*
	Não	4.631	56,3	27,6 (24,4–31,2)	
Perda dentária	≥1 dente	1.146	13,5	53,7 (45,6–61,7)	<0,001*
	Não	6.895	86,5	34,6 (30,9–38,5)	
Dor de dente	Sim	1.708	21,2	76,1 (69,0–82,0)	<0,001*
	Não	6.289	78,8	26,5 (23,3–29,9)	
Necessidade de prótese	Sim	836	9,3	67,2 (57,8–74,4)	<0,001*
	Não	7.205	90,7	34,1 (30,5–38,0)	
Sangramento gengival	Sim	2.707	34,4	46,3 (40,9–51,8)	<0,001*
	Não	5.334	65,6	32,4 (27,9–37,3)	
Presença de cálculo	Sim	2.940	35,3	42,8 (37,4–48,4)	0,003*
	Não	5.101	64,7	34,2 (30,0–38,6)	

Teste de independência com correção de Rao-Scott para amostras complexas; Amostra: número absoluto (não ponderado) de indivíduos por categoria;

* p<0,05: considerado significativo; IC95%: intervalo de confiança de 95%; OIDP: *Oral Impacts on Daily Performances*; SM: salário-mínimo.

Na Tabela 1, observa-se o comprometimento na qualidade de vida em 76,1% daqueles com odontalgia relatada nos últimos seis meses, em 67,2% dos que necessitavam de prótese dentária, em 53,7% dos que apresentavam ao menos um dente perdido, em 49,6% daqueles com um ou mais dentes cariados, em 46,3% dos que apresentavam sangramento gengival, e em 42,8% daqueles com presença de cálculo dental. Também foi verificado maior comprometimento na qualidade de vida entre os indivíduos com renda familiar de até um salário-mínimo (46,4%).

A Tabela 2 traz a associação entre agravos em saúde bucal e as atividades diárias dos adolescentes. Observa-se que ter um ou mais dentes cariados, um ou mais dentes perdidos, relato de dor de dente e necessidade de prótese foram associados às três dimensões avaliadas, físicas/funcionais, psicológicos/emocionais e sociais; enquanto a presença de cálculo dental não apresentou associação com nenhuma dimensão.

**Tabela 2:** Associação entre cárie dentária, dente perdido, dor de dente, necessidade de prótese, sangramento gengival e presença de cálculo nas atividades diárias em adolescentes brasileiros de 15 a 19 anos.

Domínio OIDP		≥1 dente cariado	≥1 dente perdido	Dor de dente	Necessita de prótese	Sangramento gengival	Presença de cálculo
		% (IC95%)	% (IC95%)	% (IC95%)	% (IC95%)	% (IC95%)	% (IC95%)
Físicos/funcionais							
	Dificuldade para comer	30,0 (25,2–35,2)*	32,4 (24,6–41,3)*	52,0 (44,1–59,9)*	46,1 (36,9–55,6)*	24,6 (20,4–29,5)**	21,3 (17,4–25,9)
	Dificuldade para falar	9,0 (6,3–12,6)*	13,6 (8,2–21,5)*	18,3 (12,7–25,7)*	18,8 (11,3–29,7)*	6,3 (4,1–9,6)	4,7 (3,3–6,7)
	Incômodo ao escovar	16,3 (12,4–21,2)	20,7 (14,5–28,9)**	16,6 (11,6–23,2)	18,7 (11,6–28,8)	14,8 (11,7–18,6)	16,2 (12,2–21,0)
	Deixou de praticar esportes	5,9 (4,4–7,9)*	9,2 (6,1–13,6)*	10,9 (7,7–15,2)*	11,3 (7,2–17,1)*	4,0 (2,8–5,5)	3,7 (2,7–5,0)
Psicológicos/emocionais							
	Sentiu-se nervoso/a ou irritado/a	25,7 (21,3–30,7)*	31,7 (24,1–40,5)*	46,6 (40,0–53,5)*	43,3 (32,5–54,9)*	19,5 (15,5–24,2)	17,6 (14,0–21,9)
	Dormiu mal/ deixou de dormir	30,0 (25,7–34,6)*	37,6 (29,5–46,4)*	52,3 (43,7–60,8)*	45,3 (35,9–55,1)*	21,9 (18,1–26,1)**	19,4 (15,9–23,6)
	Sentiu vergonha de sorrir/falar	21,0 (16,8–25,8)*	18,7 (12,9–26,2)	33,1 (27,0–39,9)*	25,5 (19,2–33,1)**	20,5 (16,7–24,9)**	19,4 (15,0–24,8)
Sociais							
	Deixou de sair/ se divertir / ir a festas	11,1 (8,6–14,3)*	16,9 (11,4–24,4)*	21,8 (17,0–27,5)*	24,0 (16,2–34,1)*	7,8 (5,9–10,2)	7,0 (5,3–9,2)
	Atrapalhou para estudar/ trabalhar / tarefas	10,6 (7,8–14,3)*	16,2 (10,5–24,1)*	20,9 (15,4–27,9)*	16,6 (10,3–25,6)*	6,1 (4,7–8,0)	6,6 (4,5–9,8)

Teste de independência com correção de Rao-Scott para amostras complexas;

* p≤0,001;

** p<0,05: considerado significativo. Cruzamento realizado entre grupo com o agravo e sem agravo; IC95%: intervalo de confiança de 95%; OIDP: *Oral Impacts on Daily Performances*.

Ter um ou mais dentes cariados foi associado (p<0,001) à dificuldade para comer (30,0%), aos distúrbios do sono (30,0%) e ao nervosismo/irritação (25,7%). A perda dentária foi associada (p<0,001) aos distúrbios do sono (37,6%), à dificuldade para comer (32,4%) e ao nervosismo/irritação (31,7%). Quanto à odontalgia, mais da metade (52,0%) dos adolescentes afetados relatou sentir dificuldades para comer e apresentou distúrbios do sono (52,3%), e 46,6% sentiram-se nervosos/irritados, com diferença significativa (p<0,001). A necessidade de uso de prótese dentária foi associada (p<0,001) à dificuldade para comer (46,1%), aos distúrbios do sono (45,3%) e ao nervosismo/irritação (43,3%). Entre os adolescentes com sangramento gengival, 24,6% relataram dificuldades para comer (p=0,005); 21,9% apresentaram distúrbios do sono (p=0,029) e 20,5% sentiram vergonha de sorrir/falar (p=0,017) (Tabela 2).

Na análise de regressão logística não ajustada, observaram-se associações entre OIDP≥1 e renda, escolaridade e agravos em saúde bucal. Adolescentes com menor renda apresentaram 2,31 vezes mais chances de relatar impacto na qualidade de vida, enquanto aqueles com menor escolaridade apresentaram 2,19 vezes mais chances. Entre os agravos, todos se associaram ao desfecho, sendo a dor dentária o fator mais expressivo, aumentando em quase nove vezes a chance de OIDP≥1. A necessidade de prótese também apresentou forte associação, com aumento de quase quatro vezes nas chances de impacto. Além disso, a presença de cárie não tratada elevou as chances em 2,57 vezes, a perda dentária em 2,20 vezes, o sangramento gengival em 1,80 vez e o cálculo dental em 1,44 vez, conforme mostra a Tabela 3.

**Tabela 3:** Modelo de regressão logística (não ajustado) entre presença de *Oral Impacts on Daily Performances* (OIDP) ≥1 com variáveis sociodemográficas e agravos em saúde bucal em adolescentes brasileiros de 15 a 19 anos.

Variável	Categoria comparada	OR	IC95%	Valor p
Sexo	Feminino *vs*. Masculino	1,27	0,99–1,63	0,055
Cor/Raça	Preta vs. Branca	1,29	0,84–1,99	0,436
	Amarela *vs*. Branca	1,05	0,51–2,18	
	Parda *vs*. Branca	1,16	0,77–1,73	
	Indígena *vs*. Branca	0,16	0,03–0,78	
Idade (anos)	17–18 *vs*. 15–16	1,02	0,74–1,39	0,595
	19 *vs*. 15–16	1,21	0,85–1,74	
Renda familiar (SM)	≤1 *vs*. >3	2,31	1,41–3,79	0,005*
	1–3 *vs*. >3	1,64	1,04–2,60	
Escolaridade	Até fundamental *vs*. Superior	2,19	1,34–3,58	0,010*
	Médio *vs*. Superior	1,56	1,01–2,43	
Cárie não tratada ≥1	Sim *vs*. Não	2,57	2,05–3,23	<0,001*
Perda dentária ≥1	Sim *vs*. Não	2,20	1,62–2,97	<0,001*
Dor nos últimos 6 meses	Sim *vs*. Não	8,85	6,48–12,10	<0,001*
Necessidade de prótese	Sim *vs*. Não	3,96	2,65–5,91	<0,001*
Sangramento gengival	Sim *vs*. Não	1,80	1,36–2,38	<0,001*
Presença de cálculo	Sim *vs*. Não	1,44	1,13–1,84	0,003*

* p<0,05 considerado significativo; OR: razão de chances (*odds ratio*); IC95%: intervalo de confiança de 95%; OIDP: *Oral Impacts on Daily Performances*.

Nos modelos ajustados, as variáveis renda e escolaridade perderam significância, porém os agravos em saúde bucal mantiveram forte associação com OIDP≥1. No Modelo 1, adolescentes com cárie não tratada apresentaram 2,39 vezes mais chances de impacto, enquanto aqueles com cálculo dental apresentaram aumento de 1,33 vez nas chances. No Modelo 2, a dor dentária permaneceu como o agravo mais fortemente associado ao desfecho, aumentando as chances em mais de nove vezes, ao passo que a perda dentária elevou as chances em 1,56. No Modelo 3, a necessidade de prótese destacou-se, aumentando em quase quatro vezes a chance de OIDP≥1, e o sangramento gengival aumentou as chances em 1,62. Os diferentes agravos bucais mantiveram associação significativa com a qualidade de vida dos adolescentes, mesmo após o ajuste por fatores sociodemográficos (Tabela 4).

**Tabela 4:** Modelo de regressão logística do *Oral Impacts on Daily Performances* (OIDP)≥1 ajustado por sexo, renda, escolaridade e agravos em saúde bucal em adolescentes brasileiros de 15 a 19 anos.

Variáveis		Modelo 1		Modelo 2		Modelo 3	
		OIDP≥1 ajustado por sexo, renda, escolaridade, cárie não tratada e presença de cálculo		OIDP≥1 ajustado por sexo, renda, escolaridade, dor e perda dentária		OIDP≥1 ajustado por sexo renda, escolaridade, necessidade de prótese e sangramento gengival	
		OR (IC95%)	Valor p	OR (IC95%)	Valor p	OR (IC95%)	Valor p
Sexo			0,064		0,093		0,014*
	Feminino *vs.* Masculino	1,28 (0,98–1,67)		1,38 (0,94–2,03)		1,55 (1,09–2,20)	
Renda (SM)			a		0,184		0,083
	≤1 *vs*. >3	a		1,63 (0,96–2,78)		1,79 (1,10–2,92)	
	1–3 *vs*. >3	a		1,29 (0,78–2,11)		1,40 (0,86–2,29)	
Escolaridade			0,055		0,190		0,081
	Até fundamental *vs*. Superior	1,76 (1,08–2,86)		1,97 (1,01–3,84)		2,30 (1,11–4,77)	
	Médio *vs*. Superior	1,33 (0,86–2,06)		1,70 (0,95–3,07)		1,84 (0,96–3,52)	
Cárie não tratada (≥1)		2,39 (1,87–3,05)	<0,001*	–		–	
Dor nos últimos 6 meses		–		9,06 (6,32–13,0)	<0,001*	–	
Perda dentária ≥1		–		1,56 (1,07–2,26)	0,019*	–	
Necessidade de prótese		–		–		3,86 (2,31–6,43)	<0,001*
Sangramento gengival		–		–		1,62 (1,14–2,29)	0,006*
Presença de cálculo		1,33 (1,03–1,71)	0,027*	–		–	

* p<0,05: estatisticamente significativo;

a Excluída após a entrada no modelo, p>0,20 na análise ajustada; OR: razão de chances (*odds ratio*); IC95%: intervalo de confiança de 95%; SM: salário-mínimo; OIDP: *Oral Impacts on Daily Performances*.

## DISCUSSÃO

Os resultados deste estudo evidenciaram associação significativa entre quase todas as variáveis de agravos em saúde bucal analisadas com a qualidade de vida dos adolescentes brasileiros. Os achados apresentaram-se em consonância com o estudo de Peres *et al.* ^27^ que, ao analisarem adolescentes de 15 a 19 anos participantes do levantamento SB Brasil 2010, identificaram que a presença de dor de dente, perda dentária e lesões de cárie não tratadas foi associada a impactos relevantes na qualidade de vida.

A dor de dente foi o agravo que mais impactou a qualidade de vida dos adolescentes, associada principalmente à alimentação, ao sono e à irritabilidade. Ela é um marcador relevante para a saúde pública, frequentemente utilizado para avaliar consequências, principalmente, da cárie dentária, sendo considerada também uma das principais motivações para a procura por atendimento e reflexo das condições de acesso aos serviços de saúde bucal^28,29^.

Uma vez que afeta diferentes aspectos relacionados à qualidade de vida de crianças e adolescentes^14^, a odontalgia é particularmente preocupante na fase escolar por associar-se ao comprometimento do desempenho e ao absenteísmo escolar, sendo considerada um indicador de sofrimento e vulnerabilidade social^30^.

A cárie dentária não tratada, comumente a causa da odontalgia, apresentou alta prevalência entre os adolescentes, impactando todas as dimensões avaliadas pelo OIDP, o que corrobora uma metanálise que evidenciou o impacto negativo de lesões de cárie nas atividades diárias de adolescentes^31^. Reforçando essa relação, uma recente revisão da literatura demonstrou que, quanto maior a gravidade das lesões cariosas, maior é o comprometimento da qualidade de vida relacionada à saúde bucal em adolescentes^15^.

Como constatado por Gerritsen *et al.* ^32^, por meio de metanálise, a perda dentária também está associada a escores desfavoráveis de QVRSB^32^, e, entre os adolescentes investigados pelo SB Brasil 2023, observou-se que a perda de pelo menos um dente foi capaz de afetar diferentes domínios relacionados ao desfecho avaliado, especialmente distúrbios do sono, dificuldade na alimentação e alterações emocionais, como nervosismo/irritação.

Em decorrência da perda dentária, observa-se que 9,3% dos adolescentes necessitavam de prótese dentária, o que, embora seja uma prevalência baixa, merece maior atenção por se tratar de indivíduos muito jovens. É fundamental reconhecer que a perda dentária ainda na adolescência constitui um problema grave, uma vez que há tendência de agravamento progressivo ao longo da vida^33^, ao se considerar a cronicidade das principais doenças bucais que acarretam a perda dentária. Importante destacar que a ausência dentária não compensada pode comprometer de maneira substancial o bem-estar físico e psicológico dos jovens, como identificado pelas análises do presente estudo, ressaltando a relevância de intervenções reabilitadoras para essa faixa etária.

Das alterações periodontais investigadas, apenas o sangramento gengival afetou as atividades cotidianas dos adolescentes, porém em menor grau comparado aos demais agravos. Alguns estudos identificaram a associação entre a presença de gengivite e cálculo dentário a impactos no sorriso, desempenho escolar e interações sociais aos 12 anos^34^, também relacionados a prejuízos na estética do sorriso aos 15 anos^25^. No entanto, Ripardo *et al.* ^35^ destacam que, embora as doenças periodontais sejam frequentes na adolescência, há baixa percepção de sua gravidade, dificultando a relação direta com prejuízos no cotidiano.

Ao ser observado um perfil de adolescentes com alta prevalência de cárie não tratada e com a QVRSB impactada principalmente pela dor de dente e necessidade de prótese, revelam-se possíveis fragilidades nas ações preventivas direcionadas a esse público, no diagnóstico precoce e na continuidade do cuidado. A ausência de tratamento em fases iniciais pode desencadear um efeito cumulativo, com agravamento da doença, maior ocorrência de dor e, em muitos casos, perda dentária, comprometendo de forma progressiva o bem-estar dos adolescentes.

Em que pese a atenção à saúde do adolescente, faz-se necessária a revisão das estratégias de cuidado, incluindo ações intersetoriais mais efetivas. O fortalecimento da atuação das equipes de saúde bucal no âmbito do Programa Saúde na Escola, por exemplo, constitui uma oportunidade estratégica para a promoção e proteção da saúde bucal, considerando-se que parte desse grupo etário ainda se encontra em ambiente escolar. A implementação de ações educativas, preventivas e de identificação precoce de agravos, alinhada às necessidades específicas dos adolescentes, pode contribuir de forma significativa para a melhoria do cenário de saúde bucal observado, com repercussões positivas sobre a qualidade de vida dessa população como consequência.

Com relação às variáveis sociodemográficas avaliadas, observou-se que as meninas apresentaram maior chance de relatar impacto negativo da saúde bucal na qualidade de vida, especialmente no modelo ajustado por necessidade de prótese e sangramento gengival. Resultados semelhantes foram identificados por Santos *et al*.^28^, em revisão sistemática com metanálise, na qual as meninas apresentaram maiores chances de relatar dor de dente, e por Militi et al.^36^, que identificaram maior impacto da aparência dentária sobre o bem-estar psicológico e na autoconfiança das meninas. Isso pode evidenciar maior autopercepção e, assim, mais chances do autorrelato do impacto da saúde bucal na qualidade de vida, especialmente durante a adolescência.

Embora as variáveis renda e escolaridade não tenham apresentado associação com o OIDP nos modelos ajustados, esse resultado não invalida a relevância social delas. A literatura demonstra de forma consistente a relação entre piores índices dessas variáveis e níveis mais baixos de literacia em saúde bucal, piores hábitos de autocuidado, menor acesso aos serviços odontológicos e maior incidência de doenças bucais^7–9,37^, sugerindo que renda e escolaridade possam atuar de maneira mais indireta na QVRSB, mediada pela presença de condições clínicas e pelo uso dos serviços de saúde. Ademais, no contexto brasileiro, a oferta de serviços públicos de saúde bucal pode atenuar parcialmente o efeito direto da renda sobre o autorrelato de impacto, sem, contudo, eliminar as desigualdades sociais subjacentes.

Este estudo destacou os impactos dos agravos bucais na qualidade de vida de adolescentes em nível nacional, mas algumas limitações devem ser consideradas. O delineamento transversal não permitiu estabelecer relações causais entre as variáveis analisadas, restringindo as inferências a associações estatísticas. Ademais, os dados sobre qualidade de vida foram obtidos por meio de autorrelato, o que pode estar sujeito a vieses de memória, sub ou superestimação de sintomas.

Dessa forma, recomenda-se que estudos futuros adotem delineamentos longitudinais, capazes de acompanhar a evolução dos agravos bucais ao longo do tempo e suas repercussões acumulativas sobre a qualidade de vida. Abordagens qualitativas podem aprofundar a compreensão das experiências subjetivas dos adolescentes diante das limitações impostas pelos problemas bucais, contribuindo para o desenvolvimento de estratégias mais sensíveis e integradas de cuidado. Investigações que incorporem determinantes sociais da saúde, como escolaridade, território, raça/cor e acesso a políticas públicas, são igualmente relevantes para o aprimoramento de ações intersetoriais voltadas à promoção da saúde bucal de adolescentes em diferentes contextos socioculturais.

Em síntese, os achados desta pesquisa ampliam a compreensão dos principais agravos que afetam a saúde bucal de adolescentes brasileiros e seus desdobramentos sobre a qualidade de vida, contribuindo para os gestores em saúde, com evidências epidemiológicas que podem subsidiar o planejamento, o monitoramento e a alocação racional de recursos, fortalecendo a vigilância em saúde bucal no âmbito da Política Nacional de Saúde Bucal. Para as equipes de saúde bucal, os achados auxiliam na identificação de agravos prioritários, qualificando a estratificação de risco, a organização da agenda e o direcionamento de ações preventivas, diagnósticas e resolutivas no território.
